# RhoGTPases and inflammasomes: Guardians of effector-triggered immunity

**DOI:** 10.1371/journal.ppat.1009504

**Published:** 2021-04-29

**Authors:** Océane Dufies, Laurent Boyer

**Affiliations:** Université Côte d’Azur, Inserm, C3M, Nice, France; University of Massachusetts, Worcester, UNITED STATES

## Abstract

Pathogens have evolved smart strategies to invade hosts and hijack their immune responses. One such strategy is the targeting of the host RhoGTPases by toxins or virulence factors to hijack the cytoskeleton dynamic and immune processes. In response to this microbial attack, the host has evolved an elegant strategy to monitor the function of virulence factors and toxins by sensing the abnormal activity of RhoGTPases. This innate immune strategy of sensing bacterial effector targeting RhoGTPase appears to be a bona fide example of effector-triggered immunity (ETI). Here, we review recently discovered mechanisms by which the host can sense the activity of these toxins through NOD and NOD-like receptors (NLRs).

## Introduction

The detection of microbes by the innate immune system is central to host immunity. Recent studies have highlighted the role of the innate immune monitoring of RhoGTPases activity to sense virulence factors targeting RhoGTPases. This is in contrast to the detection of microbial-associated molecular patterns (MAMPs) via pattern recognition receptors (PRRs) which monitors the structural motifs of microbes [1]. The sensing of virulence factors targeting RhoGTPases is based on the detection of the abnormal activity of the host RhoGTPases. This feature is related to effector-triggered immunity (ETI) that initially emerged from phytopathology studies [2,3]. Both systems play critical roles: While the sensing of microbial structural motifs expressed by most microbes would enable to sense all kinds of microbes, the detection of microbial virulence factors expressed by pathogens would enable an increased immune response specifically against pathogens.

ETI was proposed to monitor the function of microbial virulence factors (i.e., effectors and toxins) by sensing their activity [3–7]. Over the last 10 years, a major contribution to the identification of ETI in animals came from the study of virulence factors targeting RhoGTPases and their interplay with innate immune sensors such as NOD and NOD-like receptor (NLR) family members.

More than 30 virulence factors target RhoGTPases [8,9]. The mammalian RhoGTPase family consists of about 20 members, and the best characterized subfamilies are Rho, Rac, and Cdc42 [10]. Rho proteins are molecular switches that control a wide range of cellular processes including inflammation, cell death as well as tissue homeostasis [11]. Mutations of RhoGTPases or dysregulation of their activities have been linked to immune deficiencies, neurological disorders, or cancers [12–14]. The targeting of RhoGTPases by virulence factors was firstly shown to confer to pathogens a selective advantage by counteracting the innate immune responses. This encompasses the inhibition of phagocytosis and migration as well as modulation of innate immune pathways [15]. Pathogens have evolved multiple strategies to manipulate the RhoGTPase cycle. These can be divided in 2 groups: the RhoGTPase-activating toxins and the toxins inactivating RhoGTPases. Here, we review the molecular mechanism by which the host immune system senses the virulence factors that target RhoGTPases and discuss the implications of these sensing mechanisms.

## RhoGTPase-targeting toxins and virulence factors: Hijacking cellular signaling

RhoGTPases are one of the preferential targets of virulence factors, probably because of their critical role in innate immune responses. RhoGTPases have been shown to be critical regulators of the innate immune response via their contribution to phagocytosis and migration as well as the production of reactive oxygen species via NADPH oxidase [16]. RhoGTPases cycle between an active GTP-bound and an inactive GDP-bound stage which is regulated by the GTPase-activating protein (GAP), guanine nucleotide exchange factor (GEF), and guanosine nucleotide dissociation inhibitor (GDI) [17].

Bacteria use 2 types of strategies to manipulate the host RhoGTPases: (1) they use virulence factors mimicking the RhoGTPases regulators (GAP, GEF, or GDI); and (2) they utilize virulence factors endowed with enzymatic activities modifying host RhoGTPases [18]. These modifications result in either activation or inhibition of RhoGTPases, both of which affect the actin cytoskeleton and the bacterial uptake by phagocytic or non-phagocytic cells. The advantages of manipulating RhoGTPases for pathogens have been extensively studied [19–21]. Here, going through the looking glass, we will describe how these manipulations of RhoGTPases are sensed by the innate immune system.

## Sensing RhoGTPase activation to trigger a transcriptional antimicrobial response

Microbial activation of RhoGTPases induces the transcription of pro-inflammatory cytokine- and chemokine-coding genes. Interestingly, the *Salmonella* virulence factor SopE, a GEF for Rac and Cdc42, has been found to activate different signaling pathways converging on gene transcription. Firstly, *Salmonella* expressing SopE and SopE2 activates JNK, p38, and Erk MAPK, leading to NF-κB activation [22]. Interestingly, by activating Rac1 and Cdc42, SopE was shown to trigger the activation of NOD1 and Rip2 which drives cytokine production [23]. More recently, SopE has been found to activate a Cdc42-Pak1 axis leading to TAK1- and TRAF6-dependent NF-κB activation [24]. Further studies would be required to determine whether these 3 pathways are interlinked or occur separately during infection. An interesting example of an antimicrobial response triggered by RhoGTPases activation is the CNF1 toxin. The CNF1 toxin of uropathogenic *Escherichia coli* is a deamidase that was shown to trigger a protective antimicrobial response by activating the Rac2GTPase, which, in turn, activates the IMD-Relish and Rip1/2 kinases-NF-κB signaling pathways in *Drosophila* and mammalian cells, respectively [4,25].

## Sensing the inactivation of RhoGTPases by the Pyrin inflammasome

**Fig 1:**
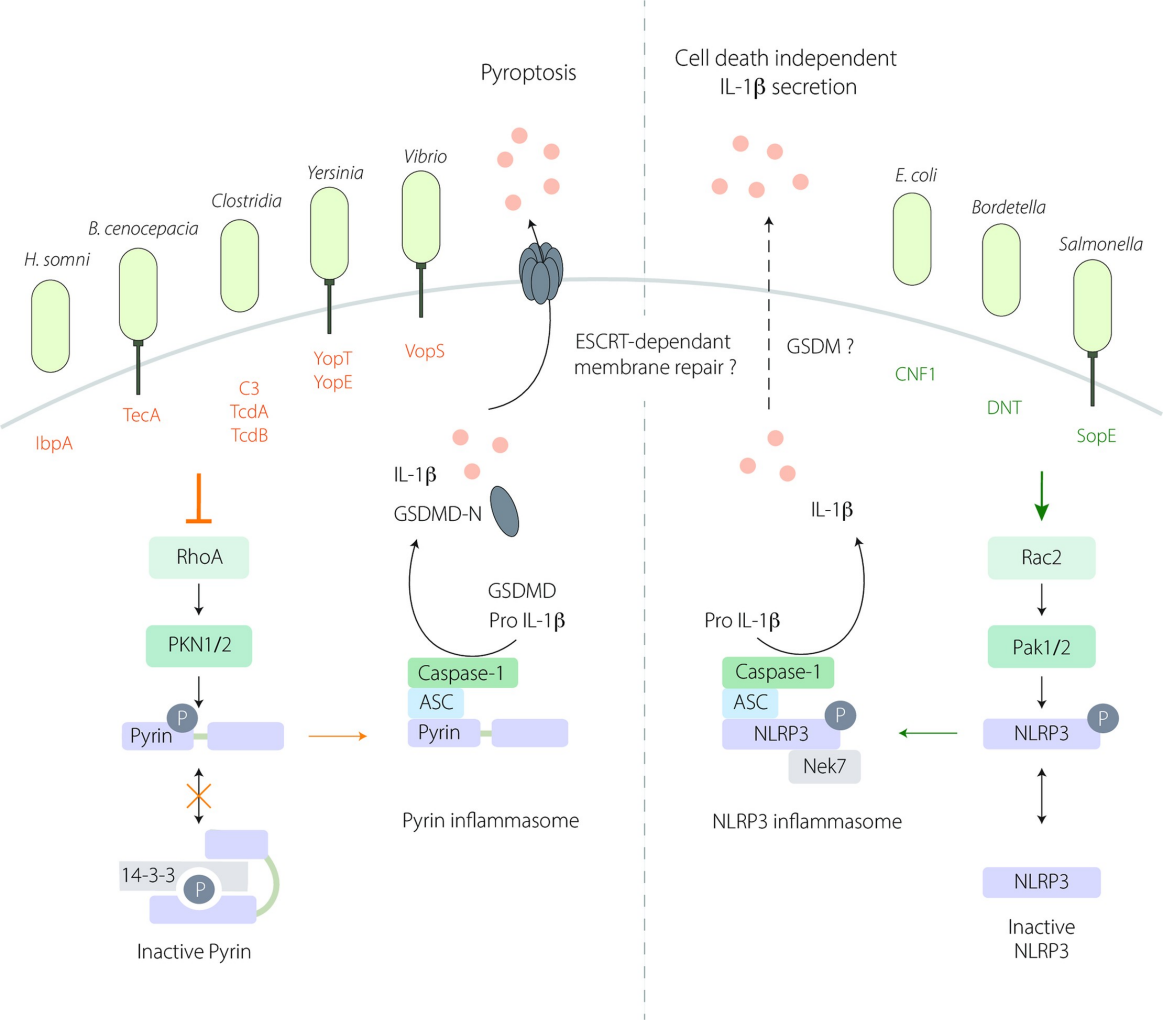
Sensing of RhoGTPase-modifying toxins by Pyrin and NLRP3 inflammasomes. (Left) The Pyrin inflammasome is activated in response to RhoA inhibition by several bacterial toxins. At steady state, active RhoA induces the activation of PKN1/2 that phosphorylates (P) Pyrin (on Ser205 and Ser241) and triggers 14-3-3—Pyrin interaction to maintain Pyrin inactive. Inhibition of RhoA by virulence factors disrupts this interaction leading to Pyrin inflammasome activation and subsequent IL-1ß maturation and GSDMD cleavage into GSDMD-N. GSDMD-N anchors to the plasma membrane and triggers IL-1ß secretion and pyroptotic cell death. The involvement of ESCRT-mediated membrane repair during Pyrin-dependent pyroptosis is not yet defined. (Right) The NLRP3 inflammasome senses Rac2 activation by bacterial virulence factors. Downstream of Rac2 activation, the Pak1/2 kinases phosphorylate (P) NLRP3 on Thr659 allowing the inflammasome assembly, and subsequent IL-1ß maturation. Is this context, IL-1ß secretion is GSDMD independent and does not trigger cell death but may involve another GSDM and/or an ESCRT-dependent membrane repair mechanism. GSDM, gasdermin; GSDMD, gasdermin D; IL, interleukin.

The *mefv* gene (coding for the Pyrin protein) was discovered through its involvement in autoinflammatory syndromes such as familial Mediterranean fever (FMF) [26,27]. Recent studies have shown a host protective function for the Pyrin inflammasome by monitoring the activity of virulence factors that inactivate RhoGTPases. The immune detection of bacterial toxins that modify the host RhoGTPases is of major importance to restrain bacterial infection. This detection system was first suggested by studies showing that TcdA and TcdB toxins from *Clostridium difficile* were able to induce Caspase-1 activation and interleukin (IL)-1ß maturation [28]. The Pyrin inflammasome was later shown by Shao and colleagues to be the sensor for RhoGTPase-inhibiting toxins. They revealed that not only TcdA and TcdB toxins but also VopS (from *Vibrio parahaemolyticus*), IbpA (from *Histophilus somni*), TecA (from *Burkholderia cenocepacia*) are detected by the Pyrin inflammasome. It is noteworthy that these toxins inactivate the RhoGTPases via 4 different mechanisms: glycosylation, ADP ribosylation, AMPylation, and deamidation [29–32]. Shao and colleagues reported that the catalytically inactive mutant of TcdB failed to activate the inflammasome, indicating the importance of sensing activity rather than conserved structural motifs. Interestingly, the toxin TcsL (from *Clostridium sordellii*) that inactivates Rac and Cdc42 but not RhoA failed to activate the Pyrin inflammasome, suggesting that Pyrin monitors the activation status of RhoA specifically [30]. It is now accepted that the Pyrin inflammasome senses the activity of RhoGTPase-inactivating virulence factors via a signaling cascade involving Ser/Thr kinases and modifications of microtubule stability. At steady state, Pyrin interacts with 14-3-3 proteins which maintain the receptor in an inactive form. RhoA inhibition by toxins results in 14-3-3 dissociation from Pyrin in a Pyrin phosphorylation status-dependent manner [31]. Park and colleagues revealed that the RhoA-interacting kinases, protein kinase N1 (PKN1) and N2 (PKN2), phosphorylate human Pyrin on Ser208 and Ser242 (Ser205 and Ser241 on murine Pyrin) and trigger 14-3-3-Pyrin interaction to maintain the inactive status of the Pyrin inflammasome [32]. Inactivation of RhoA by bacterial toxins abolishes the Pyrin phosphorylation on Ser205/Ser241 by PKN1/2 and the subsequent 14-3-3 interaction and, as a consequence, activates the Pyrin inflammasome and triggers IL-1ß secretion [31,32] (Fig 1). Interestingly, macrophage infection by *C*. *difficile*, expressing TcdA and TcdB, triggers pyroptotic cell death in a GSDMD-dependent manner [33]. Other virulence factors inactivating RhoGTPases have been shown to be sensed by the Pyrin inflammasome (Table 1). Murine macrophages infected with *Yersinia pseudotuberculosis* expressing YopE and YopT, 2 virulence factors inhibiting RhoA, trigger Ser205 dephosphorylation of Pyrin and IL-1ß secretion [34]. *Yersinia* provides a striking example of the virulence factor interplay that probably resulted from host–pathogen coevolution. Indeed, *Yersinia* injects the effector YopM that binds and activates PKN1/2 and RSK kinases to trigger Pyrin phosphorylation, thus preventing Pyrin inflammasome activation and thereby counteracting the Pyrin sensing of RhoGTPase-inactivating toxins [35–37]. Interestingly, the YopO virulence factor (YpkA in *Yersinia pestis*) has a RhoGDI domain that inhibits RhoA as well as Rac [38,39]. Further studies would determine whether it could participate to this interplay by triggering the activation of Pyrin. Another step in this host–pathogen coevolution process is the selection of human Pyrin mutations. These mutations render Pyrin insensitive to YopM and may have been evolutionarily selected to resist infection by *Y*. *pestis*, the causative agent of the plague [36]. In individuals carrying activating Pyrin mutations, the increased activity might have conferred a selective advantage against pathogens [40]. However, these mutations are also responsible for Pyrin-dependent autoinflammatory disorders [32]. The microtubule dynamics also play a role in the Pyrin inflammasome activation. Microtubules act downstream of Pyrin dephosphorylation and dissociation from 14-3-3 proteins [31,41]. Despite the clinical importance of drug targeting microtubules (such as colchicine) in Pyrin-dependent autoinflammatory disorders, the mechanisms involved in microtubule regulation of the Pyrin inflammasome are not fully understood.

**Table 1:** Inflammasome sensing of RhoGTPase-targeting by bacterial toxins.

	Toxin	Pathogen	Host target	Modification	Reference
Pyrin inflammasome	**RhoGTPase-inactivating toxins**				
	C3	*Clostridium botulinum* *Clostridium limosum* *Bacillus cereus* *Bacillus thuringiensis*	Rho	ADP ribosylation	Xu et al. (2014) [30]
	TcdA	*C*. *difficile*	Rho, Rac, Cdc42	Glucosylation	Gao et al. (2016) [31]
	TcdB	*C*. *difficile*	Rho, Rac, Cdc42	Glucosylation	Xu et al. (2014) [30]
	VopS	*V*. *parahaemolyticus*	Rho, Rac, Cdc42	AMPylation	Xu et al. (2014) [30]
	IbpA	*H*. *somni*	Rho, Rac, Cdc42	AMPylation	Xu et al. 2014 [30]
	TecA	*B*. *cenocepacia*	Rho, Rac, Cdc42	Deamidation	Aubert et al. (2016) [29]
	YopT	*Y*. *pestis* *Y*. *pseudotuberculosis* *Yersinia enterocolitica*	Rho, Rac, Cdc42	CAAX cleavage	Medici et al. (2019) [34]
	YopE	*Y*. *pestis* *Y*. *pseudotuberculosis* *Y*. *enterocolitica*	Rho, Rac, Cdc42	GAP	Medici et al. (2019) [34]
NLRP3 inflammasome	**RhoGTPase-activating toxins**				
	CNF1	*E*. *coli*	Rho, Rac, Cdc42	Deamidation	Dufies et al. (2021) [47]
	DNT	*Bordetella pertussis* *Bordetella parapertussis* *Bordetella bronchiseptica*	Rho, Rac, Cdc42	Transglutamination	Dufies et al. (2021) [47]
	SopE	*Salmonella* spp.	Rac, Cdc42	GEF	Dufies et al. (2021) [47]

GAP, RhoGTPase-activating protein; GEF, guanine nucleotide exchange factor.

## Sensing of RhoGTPase activation through the NLRP3 inflammasome

In 2004, Tschopp and colleagues established that NLRP3 is able to assemble into an NLRP3-ASC-Caspase-1 inflammasome that is responsible for autoinflammatory disorders [42]. Further studies reveal that NLRP3 inflammasome plays a role in metabolic diseases such as diabetes, atherosclerosis, and gouty arthritis [43–46]. It is thought that the main physiological function of NLRP3 inflammasome is to sense pathogen- and metabolic-triggered danger signals. The contribution of the NLRP3 inflammasome in protecting the host against infectious agents has recently emerged. The NLRP3 inflammasome is activated by several triggers such as pore-forming toxins, extracellular ATP, crystalline structures, and mitochondrial damage, but its role in ETI only recently emerged from study of RhoGTPase-activating toxins [45,47–50]. NLRP3 is regulated by phosphorylation and ubiquitination, which control its stability and subcellular localization, conformational changes, and its interaction with inflammasome-related proteins such as the adaptor protein ASC and the regulator protein Nek7 [51]. Nek7 interacts with the carboxyl terminus leucin-rich repeat (LRR) of NLRP3 and triggers a conformational change that is essential for NLRP3 oligomerization and inflammasome assembly [52–54].

The CNF1 toxin is a bona fide RhoGTPase-activating toxin encoded by uropathogenic *E*. *coli*. Interestingly, CNF1 has been shown to trigger an inflammatory immune response in vivo and in cellulo using multiple models including *Drosophila*, mouse, and human [4,25,55,56]. This CNF1-triggered protective immunity and induced bacterial clearance was observed during both *Drosophila* systemic infections and during mice bacteremia. While the CNF1-immune response in *Drosophila* was restricted to the transcriptional antimicrobial peptide expression, the CNF1-expressing *E*. *coli* triggered response in mice depended on Caspase-1 and IL-1 signaling [4,25]. Both CNF1 toxin and SopE virulence factor activating RhoGTPases have been reported to trigger Caspase-1 activation and IL-1ß maturation and secretion, but the inflammasome involved was only recently identified. The NLRP3 inflammasome has been found to be the sensor of RhoGTPase activation induced by CNF1, SopE, and DNT toxins [47] (Table 1). The NLRP3 inflammasome specifically senses the activation of Rac2. Downstream of Rac2 activation, the p21-activated kinase (Pak) 1, and Pak2 are necessary for NLRP3 inflammasome sensing of CNF1, SopE, and DNT activities. The kinases Pak1/2 regulation of NLRP3 inflammasome activation was dependent on K^+^ efflux and occurred through NLRP3 phosphorylation on Thr659 which enables the recruitment of Nek7 and inflammasome assembly and activation. During mice infections, both NLRP3 and Pak1 chemical inhibition or gene knock-out prevented CNF1-induced bacterial clearance during bacteremia. In contrast to other NLRP3 inflammasome activators, CNF1-triggered IL-1ß secretion was GSDMD independent and did not induce pyroptotic cell death (Fig 1). Further studies will be necessary to determine the mechanisms involved in IL-1ß secretion downstream of toxin-induced RhoGTPase activation. An exciting possibility would be the involvement of other GSDM in the CNF1-triggered IL-1ß secretion coupled with a cell death inhibition mechanism to control the balance between cell death and inflammation as described for GSDMD and ESCRT machinery that controls membrane repair [57,58]. In human monocyte-derived macrophages, the sensing of the CNF1 toxin via the Pak/NLRP3 axis is conserved. However, further studies are needed to determine the role of Pak and NLRP3 during infection in humans and whether Pak/NLRP3 signaling axis deficiencies are associated with increased susceptibility to infection. The NLRP3 sensing of virulence factors activating RacGTPases could better explain the coexistence within the same bacteria of virulence factors with antagonistic activities toward RacGTPases such as in *Salmonella* with SopE and SptP, respectively, GEF and GAP for RacGTPases [59].

## Concluding remarks

Pyrin and NLRP3 inflammasome guarding of RhoGTPases share striking molecular similarities such as the involvement of Ser/Thr kinases and precise phosphorylation sites to control inflammasome activation. Interestingly, PKN phosphorylation inhibits the inflammasome, while Pak phosphorylation activates the inflammasome. The fine-tuning of these Rho-regulated innate immune sensing mechanisms is probably essential for the host in order to cope with microbial infection and inflammation. The reason why the host innate immune system uses 2 different inflammasomes to monitor RhoGTPase activity remains an open question. The only easy answer is that the guarding of RhoGTPases is critical for host survival during infection.
